# Metformin as an adjuvant in breast cancer treatment

**DOI:** 10.1177/2050312119865114

**Published:** 2019-07-16

**Authors:** Mohsin HK Roshan, Yan K Shing, Nikolai P Pace

**Affiliations:** 1 Centre for Molecular Medicine and Biobanking, Faculty of Medicine and Surgery, University of Malta, Msida, Malta; 2 Pamela Youde Nethersole Eastern Hospital, Hong Kong

**Keywords:** Breast cancer, type 2 diabetes, metformin, insulin

## Abstract

Breast cancer is one of the most common malignancies in females. It is an etiologically complex disease driven by a multitude of cellular pathways. The proliferation and spread of breast cancer is intimately linked to cellular glucose metabolism, given that glucose is an essential cellular metabolic substrate and that insulin signalling has mitogenic effects. Growing interest has focused on anti-diabetic agents in the management of breast cancer. Epidemiologic studies show that metformin reduces cancer incidence and mortality among type 2 diabetic patients. Preclinical in vitro and in vivo research provides intriguing insight into the cellular mechanisms behind the oncostatic effects of metformin. This article aims to provide an overview of the mechanisms in which metformin may elicit its anti-cancerous effects and discuss its potential role as an adjuvant in the management of breast cancer.

## Introduction

Breast cancer (BC) is one of the most frequently occurring cancers in females and represents a significant public health concern. The American Cancer Society estimates that one in eight females will develop BC at some point in their lives, with the incidence increasing with age.^1^ Significant geographic and ethnic-specific differences in both incidence and mortality rates are reported. This is in part attributed to sociodemographic factors which influence adherence to recommendations for early screening for BC, as well as the likelihood of seeking appropriate medical advice upon detection of a breast mass.^2,3^ The aetiology behind BC is equally complex and involves interactions between environmental, lifestyle and genetic factors that collectively determine cancer risk.

BC typically arises when cells lose the ability to halt the process of proliferation, coupled to resistance or reduction in the process of cell death by apoptosis. BC cells express high levels of phosphatidylinositol-3-kinase (PI3K)/Akt and mammalian target of rapamycin (mTOR) signalling molecules, which impairs their ability to undergo apoptosis.^4^ Pathologically, BC is classified either as invasive or non-invasive type. The non-invasive subtypes include ductal and lobular carcinoma in situ, whereas, ductal and lobular carcinomas are considered as invasive subtypes. On average, ductal carcinoma accounts for 80% of reported cases in females, whereas lobular carcinoma accounts for only 5%–10% of the cases.^5^ Currently, BCs are treated either surgically or via chemoradiotherapy, in addition to the use of Trastuzumab (Herceptin^®^) for HER2^+^ tumours.^6^

Some cases of BC do not respond well to traditional treatment, particularly in diabetics. Recent studies have shown that metformin, a primary anti-diabetic agent, confers anti-tumorigenic effects on cancer cells and can be considered as a potential adjuvant in the management of BC.^7^ A number of population-based observational studies had initially suggested that metformin reduces cancer incidence and/or mortality among type 2 diabetic patients, however, no causal relationship can be established from epidemiologic data alone.^8^ Preclinical research using both BC cell lines and mouse models subsequently showed that metformin represses cancer cell and xenograft growth.^9–11^ These effects are achieved through various mechanisms, including cell cycle arrest, apoptosis, AMP-activated protein kinase (AMPK) activation and mTOR inhibition. In addition, metformin exerts in vitro chemo preventive effects through modulation of cytochrome P4501A1 (CYP1A1)/aryl hydrocarbon receptor (AhR) pathways.^12^ The anti-tumour effects of oral anti-diabetic therapy are not restricted to metformin, as thiazolidinediones (synthetic ligands of peroxisome proliferator-activated receptors γ – PPARγ) possess similar properties. Animal studies have shown that pioglitazone inhibits chemical carcinogenesis in rats.^13^ Human studies demonstrate that rosiglitazone reduces BC risk in females with type 2 diabetes, and that this effect is enhanced by metformin.^14^ The pleiotropic oncostatic effects of oral anti-diabetic drugs is reinforced by meta-analysis showing that thiazolidinediones are associated with a lower incidence of cancer, particularly colorectal and breast tumours.^15^

Furthermore, metformin also decreases the development of resistance in BC cells, thereby allowing current chemotherapy agents to work synergistically with metformin.^16^ It also blocks the two cellular pathways for nicotinamide adenine dinucleotide (NAD^+^) regeneration, which then results in a complete loss of cells’ NAD^+^ recycling capacity. The resulting depletion of NAD^+^, in turn, leads to cell death.^17^ This article aims to provide an overview of the pathomolecular mechanisms in which metformin may elicit its anti-cancerous effects and discuss its potential role as an adjuvant in the management of BC.

## Pathophysiology of BC

### Inflammation and neoplastic transformation in BC

The importance of the immune response in BC development and progression has been well documented. DeNardo and Coussens^18^ highlight a possible immunological connection between BC and Th2 inflammatory cells that results in the promotion of tumour development and disease progression, whereas acute anti-tumour responses involving cytolytic T lymphocytes appear to protect against tumour development. Physiologically, injured tissues or cells exposed to chemical irritants are eliminated by apoptosis. This is followed by enhanced cell proliferation to facilitate tissue regeneration and re-establish tissue function. Moreover, proliferation and inflammation may persist until the insulting agent is removed, allowing the tissue to heal completely. If the inflammation persists, cells may undergo dysplastic changes, which then increases the risk of neoplasia.^19^

The role of leukocytes, especially the cytotoxic T lymphocytes in tumorigenesis, has been explored extensively. These cells are believed to assist in the eradication process of neoplastic cells with the help of natural killer (NK) cells.^20^ Nevertheless, T-cell infiltration in invasive BC has been reported, especially the activated CD4^+^ Th1 polarised cells that secrete several inflammatory cytokines – including IFNγ, transforming growth factor beta (TGF-β), tumour necrosis factor alpha (TNFα) and interleukin-2 (IL-2). These cytokines then interact with other cytotoxic T-cells and upregulate the MHC class I and II molecules, as well as other antigen display co-factors in neoplastic cells.^18,20^ This process is an essential part of immune-mediated anti-tumorigenic effects. Conversely, activation of Th2-polarised CD4^+^ T-helper cells results in expression of inflammatory cytokines (IL-4, IL-5, IL-6, IL-10, and IL-13), which then enhances humoral immunity and downregulates cell-mediated anti-tumour immunity; thereby, promoting the pro-tumour humoral response.^18,21–24^

### Neoplastic transformation is a complex multistage event

BC originates in the undifferentiated lobules type 1, which are composed of three cell types: the highly proliferating cells (ER^−^), non-proliferating cells (ER^+^) and very few ER^+^ cells that proliferate. Endogenous 17 beta-oestradiol (E2), when metabolised by cytochrome P450 enzymes may also act as a carcinogen which ultimately leads to genomic changes and transformation phenotypes observed in spontaneously developing primary BCs. Endogenous E2 is metabolised by P450 cytochromes that also activate benzo[a]pyrene B[a] a carcinogen present in cigarette smoke.^25^ The genomic alterations induced by E2 and B[a]P in vitro are also observed in ductal hyperplasia DCIS and invasive ductal carcinoma.

Transcriptional repressors, such as Polycomb Group Protein (EZH2), which traditionally controls the cellular memory have been linked to cancer cell invasion and BC progression. Kleer et al.^26^ demonstrated that EZH2 protein levels were strongly associated with BC aggressiveness. Moreover, EZH2 overexpression promoted anchorage-independent growth and cell invasion through the SET domain and histone deacetylase activity. Dysregulated cellular memory, transcriptional repression and neoplastic transformation are interlinked, and EZH2 may be a marker for aggressive BC and neoplastic transformation. The actual neoplastic transformation process involved in BC is more complex than previously thought and warrants more long-term molecular studies to better understand the actual transformation process and ways to halt such process.

## Type 2 diabetes mellitus and cancer

Type 2 diabetes mellitus (T2DM) is a metabolic disorder which is associated with several cancers. It is characterised by hyperglycaemia, insulin resistance and hyperinsulinemia. These factors interact to promote cell proliferation through the mitogenic effect of the insulin receptor and insulin-like growth factors (IGFs), while hyperglycaemia provides the metabolic substrate for cell proliferation.^27^ Overexpression of the insulin growth factor receptor-1R (IGF-1R) or insulin receptors leads to mitogenic signalling, which increases activation of phosphoinositide 3-kinase (PI3)-Akt-mTOR signalling pathways. Excess adiposity increases local production of oestrogen via the enhanced activity of aromatase, which augments oestrogen receptor alpha signalling (ER-α) in tumour cells. The inflammatory effect of hyperinsulinemia, in addition to increasing production of local cytokines, may lead to an increased susceptibility to cancer development in diabetes.^28^

A number of large-scale epidemiological studies and meta-analyses have reported an increase in the incidence of several cancers in T2DM.^29,30^ A population-based cohort study by Ballotari et al.^31^ showed a higher cancer incidence in subjects with diabetes. This relationship was only observed in those with T2DM, but not in Type 1 diabetes mellitus (T1DM) and was attributed to obesity. Notably, the risk was higher among insulin users. An increased risk of cancer at several tissues, including liver, pancreas, endometrium, colorectum, breast and bladder has been described in multiple similar studies. Notably, these observations could be either causal – driven by the metabolic disturbances in diabetes or else due to the confounding effects of the underlying excess adiposity in diabetes. Tsilidis et al.^29^ show that individual studies are, however, characterised by substantial heterogeneity, small study effects and excess significance, with 28% (135/474) of studies adjusting risk estimates either for age or gender. Despite the evidence from epidemiological studies linking diabetes to cancer incidence, the specific mechanisms driving this association are not fully understood.

## T2DM and BC

### Mechanisms behind T2DM and BC

Studies have shown that BC in women with diabetes is often diagnosed at an advanced stage compared with women without diabetes.^32,33^ Furthermore, the overall mortality following BC diagnosis is 30%–60% higher in women with diabetes compared with women without diabetes after adjusting for tumour stage.^34,35^ A cross-sectional study by Bronsveld et al.^34^ also showed no relation between diabetic status and tumour morphology and grade. However, premenopausal diabetic women tended to develop breast tumours that do not express progesterone receptor and HER2, which are typically associated with poor prognosis. No association between insulin therapy and clinicopathological subtypes was noted, even though insulin use in T2DM may induce oestrogen (ER) and progesterone receptors expression.^36^ Conversely, a systematic review of in vitro, animal and human studies found no evidence of increased BC risk with commercially available insulin analogues and human insulin.^37^ Conflicting findings were reported by other investigators. Tseng,^38^ showed that prolonged use of insulin carries a significantly higher BC risk. A recent study by Overbeek et al.^39^ showed that females with T2DM were at an increased risk of being diagnosed with a more aggressive type of BC than non-T2DM females. Interestingly, exogenous insulin administration was not associated with the development of more advanced BC tumours in this study. These findings suggest that insulin may not be involved directly in the development of BC. Instead, it may promote BC progression by upregulating mitogenic signalling pathways.^37^

The precise mechanisms linking T2DM to BC progression remains uncertain, but is believed to involve insulin-like growth factor-1 (IGF-1). IGF-1 pathways are activated by a high concentration of insulin, which then goes on to promote cancer development via the insulin/IGF-1 hybrid receptors. These have a higher affinity for IGF-1 than for insulin and are overexpressed in BC tissues of T2DM patients.^40–42^ Nevertheless, due to insufficient evidence on the specific oncogenic pathways connecting hyperinsulinemia to BC, it is difficult to ascertain the role of insulin in the development of BC in premenopausal and postmenopausal diabetic females.

### Oestrogen, diabetes and BC

Epidemiological and clinical studies have shown that T2DM is a risk factor for BC and is consequently associated with poor prognosis.^43^ Wairagu et al.^44^ investigated the effects of oestradiol on MCF-7 BC cells primed with and without insulin chronically. The study found that insulin priming was a prerequisite for oestradiol-induced growth in BC cells. The authors demonstrate that oestradiol exposure increases expression of cyclins A and B, which are both involved in cell cycle progression and leads to the activation of genes in the pentose phosphate and serine biosynthesis pathways. Oestradiol also increased anti-apoptotic Bcl-xL expression in the insulin-primed cells. In addition, metformin suppresses oestradiol-induced growth in the insulin-primed cells. Critically, this study showed that insulin priming dramatically sensitises BC growth to 100 pmol of oestradiol.

Conversely, other studies have shown that at least 10–100 nM of oestradiol concentration is required before maximum cell growth is attainable in BC cells.^45,46^ These findings suggest that insulin priming happens readily in diabetics as a result of the chronic hyperinsulinemic state even at physiological levels of oestradiol, thus exposing diabetics to an elevated risk of developing BC. Oestradiol modulates cell cycle and apoptotic processes in insulin-primed cells, which then further promotes cancer cell growth. Wairagu et al.^44^ also showed that both insulin-primed and unprimed MCF-7 cells exposed to dihydrotestosterone (DHT) exhibit no growth response, which further indicates that there is crosstalk between insulin priming and ER-induced BC cell growth.

In the insulin resistant state, suppression of sex hormone binding globulin (SHBG) increases the bioavailability of free oestrogen, leading to elevated levels of serum oestrogen.^47^ Moreover, IGF-1 is known to increase the production of androgens, which may then subsequently displace oestrogen binding from SHBG.^48,49^ Furthermore, IGF-1 can interact with 17-beta-oestradiol leading to increased proliferation of BC cells.^50^ Therefore, altered levels of endogenous oestrogen may contribute to the proliferation of ER-positive BC in T2DM.^42^ Since the prevalence of obesity is high in T2DM, elevated levels of oestradiol and oestrone can result from increased adipose tissues aromatase activity.^51^ Also, hyperinsulinemia in T2DM may induce the expression and binding capacity of ER, which can subsequently enhance insulin mitogenic properties by promoting IRS-1 function, and through activation of PI3K and Ras/MAPK signalling.^52^ The production of inflammatory mediators in T2DM, mainly TNF-α and interleukin-6 (IL-6), which are both associated with insulin resistance in diabetics, secondarily enhances the oestrogen synthesis in normal and BC cells. This further potentiates BC development.^53,54^

### Oxidative stress, diabetes mellitus and BC

Hyperglycaemia induces oxidative stress through direct or indirect pathways in BC cells by increasing levels of insulin/IGF-1 and inflammatory cytokines, particularly IL-6 and TNF-α.^55^ Together, they activate nuclear factor kappa (NFκB), signal transducer activator of transcription 3 (STAT3) and the hypoxia-inducible factor 1-alpha (HIF1α).^56^ These factors result in increased free radical production, leading to damage to DNA, lipids and further amplification of the inflammatory processes [27]. The reactive oxidative species derived may then initiate carcinogenesis by modifying the apoptotic responses, as well as disrupting cell anchoring sites and increasing angiogenesis.^57,58^ In addition, studies have shown that hyperglycaemia also indirectly activates endothelial growth factor receptor (EGFR) via the Rho family GTPase Rac1 and cell division control protein 42 homolog (Cdc42), which then stimulates the cell proliferation, thus providing another mechanistic link between hyperglycaemia and tumorigenesis.^59^

Hyperglycaemia leads to the modulation of various pathways that control cell proliferation, migration and invasion.^60^ Cancer cells demonstrate enhanced glucose uptake and metabolism, a process referred to as the ‘Warburg effect’. Hyperglycaemia thus provides the necessary fuel which the cancer cells require, and this then allows cancer cells to proliferate rapidly.^61^ Hyperglycaemia also stimulates upregulation of protein kinase C (PKC), PPARs and proliferation in MCF-7 BC cell lines.^62^

Hyperglycaemia also promotes BC cell migration via zinc and its transporters (ZRT/IRT-like protein 6, ZRT/IRT-like protein 10). High serum glucose leads to increased expression of zinc transporters (ZIP6 and ZIP10), which are essential for promoting cell migration and motility in BC cells.^63,64^ These findings emphasise the importance of stringent control of glucose levels in both T2DM and BC in order to reduce cancer cell proliferation.

The pharmacologic management of hyperglycaemia hinges on the use of sulphonylureas, metformin and insulin. Therapy that leads to hyperinsulinemia, such as sulphonylureas and exogenous insulin, are thought to increase the risk of cancer, while treatment that reduces insulin resistance, such as metformin, are thought to reduce the risk of cancer development. A meta-analysis investigating cancer risk associated with metformin and sulphonylureas in T2DM showed that use of metformin was associated with significantly decreased risk of all cancers. However, no evidence that use of metformin is associated with the risk of BC was derived.^65^ This meta-analysis was characterised by extensive between-study heterogeneity and evidence of publication bias with regard to metformin. Hence long-term randomised double-blinded clinical trials are required to substantiate the benefit and efficacy of using anti-diabetic agents in BC treatment.

## Metformin and BC

### Mechanisms of metformin action in normal cells

Metformin (1,1-dimethyl biguanide) is a biguanide which acts by reducing hepatic glucose output and increasing insulin sensitivity. This results in a reduction in serum glucose levels without the risks of either hypoglycaemia or weight gain. Metformin also modulates multiple components of incretin pathways. It increases the plasma levels of glucagon-like peptide 1 (GLP-1) and induces islet incretin receptor gene expression via PPAR-α.^66^ Metformin is taken up by hepatocytes via the organic cation transporter 1 (OCT1) and inhibits hepatic gluconeogenesis by modulating enzymes and substrate which are involved in the gluconeogenic pathways.^67–70^ The decrease in hepatic glucose production results in the activation of AMPK, which is a cellular metabolic sensor responsible for protecting cellular functions under low energy conditions.^71,72^ AMPK is normally activated by an increase in the intracellular AMP: ratio, which results from an imbalance between the ATP production and consumption.^71^

Upon activation, phosphorylation of AMPK by tumour suppressor serine/threonine kinase 11 (STK11/LKB1) and calcium/calmodulin-dependent protein kinase kinase-2 (caMKK-2) causes AMPK to switch cells from an anabolic to the catabolic state. In doing so, it shuts down the ATP-consuming pathways by inhibiting glucose, lipid, protein synthesis and cellular growth, whereas fatty acid oxidation, as well as glucose uptake, is stimulated to restore the AMP:ATP ratio.^71^ Metformin primarily acts on the mitochondria by inducing mild and specific inhibition of mitochondrial respiratory-chain complex 1 (MRCC1), which is present in hepatocytes, skeletal muscles, endothelial cells, pancreatic beta-cells and neurons.^73–79^ In addition, metformin also reduces mitochondrial reactive oxygen species (ROS) production by selectively inhibiting reverse electron flow through MRCC1.^80,81^

ROS are important mediators of cell and genomic damage and play essential roles in the pathophysiology of both T2DM and BC. Inhibition of ROS generation through metformin may thus have benefits that extend beyond its traditional use as an oral hypoglycaemic agent. In this context, several studies have shown that metformin exhibits anti-cancer effects in BC patients with diabetes. Conversely, the efficacy of metformin and its use in non-diabetic BC patients is not widely studied, with conflicting effects being reported. A summary of the findings from studies involving metformin therapy in BC patients without DM is provided in Table 1.

**Table 1.** A summary of the salient findings from studies investigating metformin administration in non-diabetic females with breast cancer.

Title	Study	Study population	Study findings	Comments
Metformin may protect non-diabetic breast cancer women from metastasis	El-Haggar et al.^82^	Non-diabetics newly diagnosed with BC	A total of 102 women newly diagnosed with BC were divided into the control group and metformin group. All women were treated with adjuvant therapy and the metformin-treated group received 850 mg of metformin twice daily. The metformin group demonstrated lower IGF-1, IGF1: IGFBP-3 ratio, insulin, FBG and HOMA-IR. Metformin had potential anti-tumour and anti-metastatic effects that require further exploration	Follow-up for 12 months only. and study did not indicate whether metformin use had affected different stages of the BC
Metformin anti-proliferative effects on a cohort of non-diabetic breast cancer patients	Sadighi et al.^83^	Non-diabetics with BC on metformin versus non-diabetics with BC not on metformin	A prospective randomised controlled study about metformin efficacy in the window time between biopsy and definite surgery were carried out. Primary endpoint was changes in Ki67 expression. The intervention group was prescribed 1500 mg/day metformin from biopsy to night before surgery. A significant reduction in Ki67 was noted. Study concluded that metformin prescription for short period exhibits an inhibitory effect on BC cell growth	The population size for the study was small (50 patients) and the time frame between metformin use and the obtained results was relatively short. Further long-term trials are needed
Insulin-lowering effects of metformin in women with early breast cancer	Goodwin et al.^84^	Early BC patients	22 non-diabetic females with BC treated with metformin (500 mg tid × 6 months) showed lower insulin levels, and it improves insulin resistance in non-diabetic females with BC. More long-term trials are recommended	
Evidence for biological effects of metformin in operable breast cancer: a pre-operative, window-of-opportunity, randomised trial	Hadad et al.^85^	Operable invasive BC	Trial examined effects of metformin on Ki67 and gene expression in primary BC in non-diabetic females. 47 patients were randomised to metformin 500 mg od for a week and increased to 1 g bd for a further week until surgery. Gene set analysis revealed p53, BRCA1 and cell cycle pathways showed reduced expression following metformin administration	Population size was small and not age-matched
Evidence for biological effects of metformin in operable breast cancer: biomarker analysis in a pre-operative window of opportunity randomised trial	Hadad et al.^86^	Operable invasive BC in non-diabetics	Non-diabetic females with operable invasive BC randomised received pre-operative metformin (500 mg daily for 1 week and then 1 g bd for a further week) and control receiving no metformin. Level of Ki67 and cleaved caspase-3 fell significantly in metformin-treated cohorts but not in control	Metformin acts on BC cells via pAMPK upregulation and downregulation of pAkt and insulin suppression through cytostatic mechanisms
Dual effect of metformin on breast cancer proliferation in a randomised pre**-**surgical trial	Bonanni et al.^87^	Operable BC	A total of 200 non-diabetic females with operable BC administered with 850 mg bd metformin (n = 100) and placebo (n = 100). Metformin prior to surgery did not affect Ki67 levels but did affect the insulin resistance in tumour cells	
The effect of metformin on apoptosis in a breast cancer presurgical trial	Cazzangia et al.^88^	Operable BC	A total of 87 females (45 on metformin, 42 on placebo) assessed for 4 weeks before surgery. No modulation of apoptosis by metformin was found but it did affect the insulin resistance status and the Ki67 levels	
Differential effects of metformin on breast cancer proliferation according to markers of insulin resistance and tumor subtype in a randomised pre-surgical trial	DeCensi et al.^89^	Operable BC	A total of 200 non-diabetic females randomly allocated to metformin (850 mg bd) or placebo for 4 weeks prior to BC surgery. Compared to placebo, metformin reduced Ki67 in women with HOMA-IR > 2.8 and HER2 positive tumours. No trend was noted in women with HOMA-IR < 2.8	Study showed that at conventional anti-diabetic dosage, the effect of metformin on Ki67 depends on the tumour characteristics and insulin resistance
Presurgical trial of Metformin in overweight and obese patients with newly diagnosed breast cancer	Kalinsky et al.^90^	Invasive BC or DCIS	A total of 1500 mg of metformin was administered to 35 non-diabetics with stages 0–III BC and BMI of >25 kg/m^2^. No reduction in Ki67 was noted after metformin administration compared to controls, but there was significant reduction in BMI, cholesterol and leptin levels	Trial registration NCT00930579
Effect of metformin vs placebo on and metabolic factors in NCIC CTG MA.32	Goodwin et al.^91^	Early BC	The NCIC Clinical trial group MA.32 investigated effects of metformin vs placebo on invasive disease-free survival and other outcomes in early BC. 492 females were randomly assigned treatment for 6 months. Study concluded that metformin significantly improved weight, insulin, glucose, leptin and hsCRP at 6 months	
Metformin in early breast cancer: a prospective window of opportunity neoadjuvant study	Niraula et al.^92^	Operable BC	A total of 39 patients with newly diagnosed, untreated, non-diabetic BC patients were treated with 500 mg tid metformin after diagnostic core biopsy until definitive surgery. Study concluded that short-term use of metformin resulted in beneficial clinical and cellular changes (reduced BMI, weight, HOMA, reduced Ki67 staining in invasive tumour) consistent with beneficial anti-cancer effect	
Changes in insulin receptor signalling underlie neoadjuvant metformin administration in breast cancer: a prospective window of opportunity neoadjuvant study	Dowling et al.^93^	Operable BC in non-diabetics	Neoadjuvant, single-arm trial conducted to determine the clinical and biological effects of metformin on patients with BC. Females with untreated BC were administered 500 mg of metformin tid for over 2 weeks from after diagnostic biopsy until surgery. Results showed a reduction in PKB/Akt and ERK 1/2 phosphorylation with decreased insulin and IR levels	Clinical trial NCT00897884
Metformin intervention in obese non-diabetic patients with Breast Cancer: phase II randomised, double-blind, placebo-controlled trial	Ko et al.^94^	Obese females with BC	The randomised, double-blinded, placebo-controlled trial performed to evaluate the efficacy of metformin for controlling physical and metabolic profile in non-diabetic BC patients. 105 females with BC at 6 months post-mastectomy with BMI of >25 kg/m^2^ and/or pre-diabetes were assigned into three groups (placebo, metformin 500 mg and metformin 1000 mg) stratified by tamoxifen use. Study found that metformin 1000 mg had favourable effects on controlling glucose and HbA_1C_ levels in obese non-diabetic BC patients	

AMPK: AMP-activated protein kinase; BC: breast cancer; IGF: insulin-like growth factors; DCIS: ductal carcinoma in situ; BMI: body mass index.

### Mechanism of metformin action in BC

Insight into the anti-tumour role of metformin in BC has been provided by Dowling et al.^93^ in a clinical trial (NCT00897884). Non-diabetic females with untreated BC were trialled on neoadjuvant metformin from biopsy till surgery for BC. Immunohistochemical analysis of tumour specimens showed a significant reduction in expression of insulin receptors, phosphorylation of protein kinase B (PKB)/Akt, AMPK, extracellular signal-regulated kinase1/2 and acetyl coenzyme A carboxylase. These insulin-dependent effects are consistent with the beneficial anti-cancer effects of metformin.

**Figure 1.**
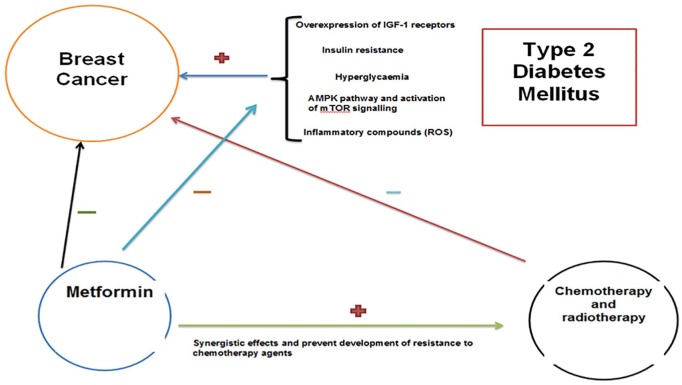
Metformin inhibits the inflammatory pathways which are induced by hyperglycaemia and insulin resistance. This indirectly acts by deactivating AMPK pathways, thereby allowing the anti-cancer effects of metformin to be exhibited. Metformin also works synergistically with chemotherapy agents and reduces the development of resistance of BC to these agents, thereby maximising their effects on BC cells.

Metformin indirectly activates AMPK, leading to the inhibition of protein synthesis and gluconeogenesis. Thus, it may act to limit the availability of nutritional substrates that are mandatory for cancer cell proliferation.^95^ An overview of these effects is provided in Figure 1. Furthermore, AMPK also inhibits mTOR, which is a downstream activator of growth factors in malignant cells and is associated with resistance to anti-cancer drugs.^95^ The role of metformin is not limited to AMPK pathways. It induces cell cycle arrest, thereby inducing sub-G_1_ populations and activating apoptotic pathways through downregulation of p53 and differentiated embryo chondrocyte 1 (DEC1) proteins.^96^ Metformin administration also leads to an increase in intracellular ROS by disrupting the mitochondrial electron transport chain and collapsing the mitochondrial membrane potential. Queiroz et al.^97^ showed that metformin has time- and concentration-dependent anti-proliferative properties on MCF-7 cells. Metformin exhibits pro-apoptotic effects and promotes cell cycle arrest via increased oxidative stress, as well as AMPK and FOXO3a activation.

The proliferation and migration of BC cells is suppressed by metformin via the dysregulation of the matrix metalloproteinases MMP-2 and MMP-9, in addition to downregulation of oncogenic microRNAs miR-21 and miR-155.^98^ Giles et al.^99^ demonstrated that metformin can decrease the size of mammary tumours and inhibit tumour formation in ovariectomised rats with 1-methyl-1-nitrosourea (MNU)-induced mammary tumours. Furthermore, metformin promotes a decrease in the number of aromatase-positive, CD68-positive macrophages within the tumour microenvironment, as well as decreased lipid accumulation in the livers of treated rats. This study showed that metformin targets both whole-body metabolism and the tumour microenvironment and that the perimenopausal period may represent a window of opportunity where metformin may be highly effective in women at risk for or with established BC. Other investigators have produced similar findings, demonstrating decreased tumour volumes and reduced proliferation in in vivo animal models of BC.^100,101^ Recently, Bojkova et al. showed that administration of metformin in a rat model with MNU-induced mammary tumours resulted in an increased proportion of low-grade tumours.^102^ A significant positive correlation between histological grade and Ki67 expression was noted. However, no differences in tumour incidence and frequency were observed. The improved tumour histopathological profile was accompanied by a reduction in serum IGF-1 levels.

Metformin also exhibits cytostatic effects analogous to antifolate chemotherapeutic agents. In vitro metabolomic studies have shown that metformin has mitochondrial-independent AMPK-activating effects that cause defects in de novo purine/pyrimidine biosynthesis and homocysteine accumulation.^103^

Metformin also exerts anti-inflammatory effects in cell models by inhibiting the NFκB pathways necessary for transformation and cancer stem cell formation. It inhibits nuclear translocation of NFκB and phosphorylation of STAT3 in cancer stem cells compared with non-stem cancer cells in the same population, thus suppressing the early stages of the inflammatory pathway that is associated with cancer.^104–106^

In light of these findings from cell and animal models, it is natural to question whether metformin is a suitable adjuvant and if it should be implemented in current clinical practice guidelines for the treatment of BC. Clinical data extracted from drug trials have shown that metformin does show synergistic apoptotic effects when used with chemotherapeutic agents in BC.^50,107^ Furthermore, when metformin is used as a single-agent, it may trigger cell cycle arrest in both oestrogen receptor positive (ER^+^) and ER-negative (ER^–^) BCs cells.^108,109^ Metformin also elicits toxic effects on cancer stem cells, but not on normal stem cells. This property of metformin is valuable since cancer stem cells play a critical role in cancer recurrence.^104,110^ A number of systematic reviews and meta-analyses highlighting metformin’s role in BC and their limitations are summarised in Table 2.

**Table 2.** Summary of systematic reviews and meta-analysis available on metformin’s role in breast cancer.

Title	Authors and date of publication	Summary of the study	Comments
The effect of metformin on biomarkers and survival for breast cancer – a systematic review and meta-analysis of randomised clinical trials	Zhang et al.^111^	Ten studies comprising 1520 BC patients. Metformin reduces levels of insulin, HOMA-IR, sex hormones, SHBG, Ki67, caspase-3, p-Akt, obesity, hs-CRP, blood glucose and lipid profile. Overall survival was non-significantly better in metformin arm than control arm. The authors concluded that further large-scale trials are needed	The authors suggested that observational studies used for the systematic review and meta-analysis had residual confounding, selection biases and warranted data from more randomised clinical trials. They concluded that current data regarding metformin in BC is lacking and inconsistent
Association of Metformin with Breast Cancer Incidence and Mortality in Patients with Type II diabetes: A GRADE-Assessed Systematic Review and Meta-analysis	Tang et al.^112^	Twelve observational studies were included for BC incidence and 11 studies for all-cause mortality. No significant association was found between metformin exposure and incidence of BC. A 45% risk reduction was observed for all-cause mortality. The use of metformin may improve overall survival in patient with T2D and BC	
Metformin as an adjuvant therapy for cancer: A systematic review and meta-analysis	Coyle et al.^113^	A meta-analysis of 27 observational studies with 24,178 subjects. No significant beneficial outcome was observed with regard to BC in metformin users compared with non-users. Significant benefit was, however, observed for early-stage colorectal and prostate cancer	This meta-analysis was not specifically exploring BC incidences, instead it was evaluating the effect of metformin on recurrence-free survival, overall survival and cancer-specific survival. Data were not available to conduct analyses on the impact of metformin dose and duration
Diabetes mellitus is associated with breast cancer: a systematic review, meta-analysis and in silico reproduction	Zhou et al.^114^	Twenty studies were used for this meta-analysis to explore the association between DM and BC survival outcome. Pre-existing diabetes was associated with 37% increase in all-cause mortality risk in females with BC	Cohorts used by the selected studies were not age-matched, nor was the dose of metformin used in each study specified
Adjuvant chemotherapy for early female breast cancer: a systematic review of the evidence for the 2014 Cancer Care Ontario systemic therapy guideline	Gandhi et al.^115^	The Program in Evidence-Based Care (PEBC) of Cancer Care Ontario complied a guideline on the systematic treatment of early BC. The Early Breast Cancer Trialists’ Collaborative Group meta-analyses included RCTs. Chemotherapy was divided into three classes: anti-metabolite-based regimens, anthracyclines and taxane-based regimens. In terms of metformin use in early BC, the review specified that this drug is still in the early investigation phase	
Metformin and cancer risk and mortality: a systematic review and meta-analysis taking into account biases and confounders	Gandini et al.^116^	A meta-analysis was carried out using 71 articles related to metformin and cancer incidence or mortality. Results showed that metformin may reduce cancer incidence and mortality in diabetics, but the reduction is modest and does not affect all populations equally	The limitations of the study included heterogeneity of study design and treatment comparators. Approximately 2/3 of the studies were prone to source of bias as they were retrospective. The comparator group included treatment with insulin and insulin secretagogues. These drugs increase levels of insulin and have been associated with increased cancer risk. Allocation bias was also reported, with metformin users being at different stage of diabetes than comparators
The prognostic value of metformin for cancer patients with concurrent diabetes: a systematic review and meta-analysis	Zhang and Li^117^	Twenty-eight studies were used for this meta-analysis. The authors showed that metformin lowers the risk of all-cause mortality in cancer patients with concurrent diabetes, particularly in BC. The findings from the study supported the notion that metformin improves the survival for cancer patients with concurrent diabetes, particularly breast, colorectal, ovarian and endometrial cancers, but warrants further investigation in relation to the efficacy	Limitation of the meta-analysis include the high heterogeneity across the studies. The information on cancer treatment was not described clearly by each of the studies that were used, thus creating a bias effect on the survival rate. Moreover, the dose of metformin and cancer stage were not reported
Metformin therapy and risk of cancer in patients with type 2 diabetes: systematic review	Franciosi et al.^118^	Twelve randomised controlled trials (21,595 patients) and 41 observational studies (1,029, 389 patients) were used for this analysis. Metformin use was associated with significantly reduced risk of some cancers (liver, colorectal, pancreas, stomach and oesophagus) and cancer-related mortality. No effect on the risk of BC was reported	The authors concluded that the results derived from the observational studies were prone to bias and confounding inherent due to their design. In addition, some studies were retrospective, and suffered from interviewer and recall bias. The data on dose, duration of therapy and variation over time for treatment, as well as full information on risk factors and potential confounders were incomplete
Metformin and breast cancer risk: a meta-analysis and critical literature review	Col et al.^119^	Seven independent observational studies were included in this systematic review. The authors showed that metformin has a protective effect on BC risk among postmenopausal females with diabetes	Results obtained were limited by the observational nature of the reports and different comparison groups that were not age-, race- or stage-matched
Metformin and cancer risk in diabetic patients: a systematic review and meta-analysis	Decensi et al.^120^	Eleven studies reporting 4042 cancer events and 529 cancer deaths were selected for this meta-analysis. A 31% reduction in overall relative risk of pancreatic and hepatocellular cancer in subjects taking metformin compared with other anti-diabetic drugs was reported. No significant findings for breast, colon and prostate cancer were reported	

BC: breast cancer; SHBG: sex hormone binding globulin; hs-CRP: high-sensitivity C-reactive protein; DM: diabetes mellitus; RCT: randomised controlled trial.

Triple-negative BC (TNBC) carries the poorest prognosis of all BC subtypes. In vitro studies have shown that metformin administration enhances the sensitivity of TNBC cell lines to TRAIL receptor agonists.^121^ TRAIL agonists (TNF-related apoptosis-inducing ligand (TRAIL) are tumour-specific inducers of apoptosis that have strong anti-tumour effects in preclinical models.^122^ Metformin reduces the levels of XIAP, a negative regulator of TRAIL-induced apoptosis, and provides evidence supporting the combined administration of these drugs. Other in vitro studies have demonstrated that metformin reduces the percentage of TNBC stem cells through mechanisms that downregulate Krüppel-like factor 5 (KLF5) and target its degradation.^123^ The downregulation of KLF5 is mediated by glycogen synthase kinase-3β (GSK3β) and inhibition of protein kinase A activity in TNBC cells. KLF5 is a crucial stem cell transcription factor in basal-type TNBC cells, and it promotes TNBC cell proliferation, survival, migration, invasiveness and stemness.^124–126^ The reduced TNBC stem cell viability observed in vitro has significant consequences which need to be evaluated further in in vivo animal models. Metformin also has been shown to downregulate fatty acid synthase (FASN) levels via miR-193b in TNBC cells. FASN is an essential component of de novo fatty acid synthesis and is thus necessary for the survival of TNBC cells.^127^

Despite the beneficial anti-tumour potential of metformin in TNBC, other studies have suggested that this effect is reduced by higher glucose concentrations.^128–130^ Recently, Varghese et al. show that TNBC cell lines exposed to glucose levels in the diabetic range significantly abolished the effect of metformin on cell proliferation, cell death and cell cycle arrest. This study also showed that metformin was most effective and inhibited the mTOR pathway under glucose starvation conditions; suggesting that it should be combined with inhibitors of the glycolytic pathway for more beneficial treatment of TNBC in diabetic patients.^129^ In view of the mechanistic evidence linking the anti-proliferative effect of metformin to glucose concentration in TNBC, it is natural to advocate stringent glucose level monitoring in BC patients with diabetes, particularly as the hyperglycaemic state may further fuel malignant cell proliferation. The anti-cancer effects of metformin are not limited to triple-negative and ER^+^ BC subtypes. Metformin is also effective against HER2^+^ BC since it confers anti-proliferative effects in females with HER2^+^ BC co-expressed with ER^+^ with ductal carcinoma in situ (DCIS).^131^ Nonetheless, the molecular mechanisms behind these findings are inadequately explained.

Clearly, the use of metformin in the management of BC requires further evaluation. Preliminary population-based studies have shown that metformin does not affect BC staging in older women with long-standing diabetes.^132^ These findings contrast with both the short-term window of opportunity studies and with functional research highlighted earlier that show an effect of metformin on tumour growth characteristics.

### Non-specific effects and limitations of metformin in BC

Like many other chemotherapy agents currently in use, the development of multidrug resistance by cancer cells proves to be a considerable challenge for clinicians. Interestingly, some studies have suggested that metformin may prevent multiple drug resistance (MDR) and may even re-sensitise cancer cells to standard chemotherapy agents to which they were once sensitive. In vitro and in vivo animal studies show that metformin reduces the expression of several proteins that cause MDR.^133^ Metformin also has MDR reversing properties in BC cell lines and re-sensitises cells to 5-fluorouracil (5-FU), adriamycin and paclitaxel through the activation of AMPL and mTOR pathways.^134,135^ In addition, it has been shown to modulate the metabolic and miRNA profile of chemoresistant cells, rendering them similar to chemosensitive BC cell populations.^136^ Other investigators have demonstrated that co-treatment of BC cells with metformin and flavone inhibits cell viability and increases apoptosis of cancer cells more effectively compared with metformin or flavone alone.^137^ This potentiation of apoptosis is achieved by the modulation of MDMX/p53 proteins through PI3K/AKT pathways.

Conversely, chronic exposure to metformin has been shown to lead to the development of resistance in BC cell lines. Acquired resistance to metformin is accompanied by transcriptomic changes that generate a metastatic stem-cell like phenotype.^138^ In addition, it has been shown that long-term treatment with metformin can lead to the development of cross-resistance to both metformin and tamoxifen in MCF-7 cells.^139^ Scherbakov et al.^117^ show that the acquired resistance to both drugs is based on the constitutive activation of Akt/Snail1/E-cadherin signalling pathways.

Why metformin confers anti-tumour effects in some, but not all BC cases is not clear. The AMPK-activating ability of metformin is central to its metabolic function in cells. Buac et al.^104^ show that breast cancer-associated gene 2 (BCA2) is an endogenous inhibitor of AMPK activation in BC cells. BCA2 encodes a RING-finger-containing ubiquitin E3 ligase that is expressed in about 50% of breast tumours. This gene has been associated with both in vitro BC cell proliferation and clinical outcome.^140^ Inhibition of BCA2 enhances the growth inhibitory effect of metformin in cell models, thus suggesting that metformin co-administration with a BCA2 inhibitor can be a more powerful strategy than metformin therapy in isolation.^141^

The dose of metformin required to achieve a therapeutic effect is similarly controversial. Several doses have been used in studies with varying clinical effects. In fact, Schexnayder et al.^142^ showed that metformin at pharmacologically achievable concentrations does not significantly improve the viability of BC cells. Instead, it inhibits inflammatory signalling and metastatic progression of the disease through reduced ICAM1, COX2, PGE2 and ROS levels. Cell cycle arrest and decreased cell viability were only reported at higher concentrations of metformin.

The mechanistic findings from preclinical in vitro research are not directly translatable to clinical practice. A recent meta-analysis of observational studies on the effect of metformin on the incidence and all-cause mortality of BC in patients with type 2 diabetes failed to identify a significant association between metformin exposure and incidence of BC, while a 45% risk reduction for all-cause mortality was observed.^112^ The uncertainty regarding the optimal dosage, duration of therapy and whether additional drugs should be co-administered with metformin to achieve synergistic effect further limits its clinical use. An ongoing phase II randomised clinical trial (NCT01589367) aims to investigate the effect of the aromatase inhibitor letrozole with metformin in postmenopausal patients with ER + BC.^143^ Further such studies are required in order to formulate guidelines to advise clinicians on the possible therapeutic implementation of metformin in BC.

This review on metformin therapy in BC has several limitations. Primarily, it does not aim to provide a systematic review of all mechanistic and epidemiologic evidence on the subject. Several authors have compiled evidence from individual studies in an attempt to resolve discrepancies and inconsistencies between different investigations, and selected key publications are summarised in Tables 1 and 2. The extent of heterogeneity and discordant findings among individual studies is significant and serves to highlight the complexity of the subject. Second, the link between metformin exposure and BC is unlikely to be a simple cause–effect relation. BC, glucose metabolism and the pharmacodynamics of metformin represent cellular events that are intrinsically heterogeneous and multidimensional and that are not fully elucidated. The interplay between each element of this complex interaction depends on various genetic, epigenetic and lifestyle factors that cannot be fully quantified and might not be faithfully reproduced in invitro preclinical studies. In the era of precision medicine and single-cell tumour biology, it is essential for researchers to acknowledge disease heterogeneity and functional diversity within solid tumours as this can significantly impact on clinical outcomes.

## Conclusion and future directions

BC is an etiologically complex devastating disease driven by a combination of genetic, reproductive, hormonal and environmental factors. The epidemiologic link between BC and disordered glucose metabolism is mechanistically interesting, given that glucose is an essential cellular metabolic substrate and that insulin signalling has mitogenic effects. The common underlying mechanism uniting T2DM and BC involves hyperinsulinemia, which activates several molecular pathways driving cell proliferation.^144^ BC has been traditionally treated with a combination of chemotherapy, surgery and targeted hormonal therapy; however, growing interest lies in the use of metformin. Metformin activates AMP-activated protein kinase and inhibits mTOR pathways, thereby decreasing insulin levels in the circulation. In addition, it also inhibits the proliferation and invasion of cancer cells, which could limit metastatic spread. Studies have also demonstrated that metformin may enable cancer cells to overcome the development of resistance to chemotherapy, hormone therapy and trastuzumab treatment.

The use of metformin in the management of both T2DM and BC may seem practical considering that metformin is one of the most commonly prescribed oral anti-diabetic agents. It accounts for 40% of all anti-diabetic drugs dispensed in England over the past few decades, but only 7% of the costs.^145^ The recent ALTTO trial on metformin use in HER2^+^ BC showed that metformin may improve the worse prognosis that is associated with diabetes and insulin treatment in patients with HER2^+^ and hormone receptor positive BC.^146^ Furthermore, promising in vivo studies suggest that metformin can have beneficial synergistic effects with natural anti-tumour compounds such as curcumin.^147^ Meta-analysis of large cohorts have demonstrated that metformin use is associated with improved survival and decreased all-cause mortality in diabetic patients with BC.^148,149^ Conversely, conflicting clinical findings with regard to the efficacy and anti-tumour role of metformin have been reported in the literature, thus strengthening the need for further research.^132,150^

The potential for therapeutic benefits of metformin in diabetics with BC is rapidly becoming an area of interest in both clinical oncology and endocrinology. However, more long-term double blinded-randomised trials are needed to explore the precise role which metformin may play and its possible use as an adjuvant in clinical practice. Most current studies that examine metformin’s use in BC have reported a mixed picture on its efficacy, which could be due to the different doses of metformin as well as varying periods of follow-up used in these studies. It is clear that metformin holds considerable promise with regard to a potential anti-tumour role.
